# In silico pharmacology: drug design and discovery’s gate to the future

**DOI:** 10.1186/2193-9616-1-1

**Published:** 2013-02-12

**Authors:** Hamid R Noori, Rainer Spanagel

**Affiliations:** Institute of Psychopharmacology, Central Institute for Mental Health, Medical Faculty Mannheim, University of Heidelberg, Mannheim, 68159 Germany

Introduction of new drugs and novel therapeutic solutions is a long and costly process (Myers and Baker 2001; DiMasi et al., 2003). Traditionally, pharmacologists strive to optimize and accelerate this process by developing new *in vivo* and *in vitro* investigation strategies.

However, the last decades have been witnessing the rise of alternative research models, the so-called *in silico* approaches, using computational environments as their experimental laboratories.

Imitating the common biological terms *in vivo* and *in vitro*, the term *in silico* refers to performing experiments using computers. Although the historical origin of this term is not clear, it is safe to assume that *silico* is a reference to the chemical element Silicon (Si), a key component of computer chips.

The majority of the *in silico* methods are primarily used in parallel with the generation of *in vivo* and *in vitro* data for accurate modeling and validation of a wide range of applications from the ligand design and optimization to the characterization of fundamental pharmacological properties of molecules such as absorption, distribution, metabolism, excretion and toxicity (Ekins et al., 2007). The diversity of the developed mathematical and biophysical models in this field resembles the manifoldness of the pharmacological problems uniquely.

While the seminal work of Hansch and Fujita (1964) on the statistical relationships between the molecular structure and a specific chemical or biological property (*Quantitative structure-activity relationships*) initiated the application of modern data mining and statistical techniques such as the *virtual ligand screening (*Oprea and Matter, 2004*)* and the *virtual affinity profiling* (O'Connor and Roth, 2005; Paolini et al*.,*^2006^), biophysical (Jones and Woodhall, 2005; Graupner and Gutkin, 2009) and neurochemical network models (Noori and Jäger, 2010; Noori, 2012; Noori et al., 2012) mainly apply deterministic dynamical systems to identify drug-induced alterations of electrophysiological and/or neurochemical network characteristics.

In light of the rapid progress of *in silico* approaches, it could be expected that biomedical investigations in virtual reality ultimately lead to rigorous changes in the pharmaceutical research landscape by optimizing the drug development process, reducing the number of animal experiments and smoothing the path to personalized medicine.

Despite the increasing interest in this field of research, publication platforms with dedicated agenda to in silico pharmacology are missing. With the launch of our journal, we aim to fill this gap and provide a forum for interdisciplinary research articles that specifically address computational approaches in drug-design and multi-scale analysis of bioactive substances from the cellular up to behavioral level.

## References

[CR1_1] DiMasi JA, Hansen RW, Grabowsk HG (2003). The price of innovation: new estimates of drug development costs. J Health Econ.

[CR2_1] Ekins S, Mestres J, Testa B (2007). In silico pharmacology of drug discovery: methods of virtual ligand screening and profiling. Br J Pharmacol.

[CR3_1] Graupner M, Gutkin B (2009). Modeling nicotinic neuromodulation from global functional and network levels to nAChR based mechanisms. Acta Pharmacol Sin.

[CR4_1] Hansch C, Fujita T (1964). Rho-sigma-pi analysis. A method for the correlation of biological activity and chemical structure. J Am Chem Soc.

[CR5_1] Jones RS, Woodhall GL (2005). Background synaptic activity in rat entorhinal cortical neurones: differential control of transmitter release by presynaptic receptors. J Physiol.

[CR6_1] Myers S, Baker A (2001). Drug discovery—an operating model for a new era. Nat Biotechnol.

[CR7_1] Noori HR, Jäger W (2010). Neurochemical oscillations in the basal ganglia. Bull Math Biol.

[CR8_1] Noori HR, Spanagel R, Hansson A (2012). Neurocircuitry for modeling drug effects. Addict Biol.

[CR9_1] Noori HR (2012). The effects of the acute administration of low-dosage ethanol on the phasic neurochemical oscillations of the basal ganglia. Math Med Biol.

[CR10_1] Oprea TI, Matter H (2004). Integrating virtual screening in lead discovery. Curr Opin Chem Biol.

[CR11_1] O'Connor KA, Roth BL (2005). Finding new tricks for old drugs: an efficient route for public sector drug discovery. Nat Rev Drug Discov.

[CR12_1] Paolini GV, Shapland RH, van Hoorn WP, Mason JS, Hopkins AL (2006). Global mapping of pharmacological space. Nat Biotechnol.

